# BARRIERS AND SOLUTIONS TO VOLUNTEERING FOR OLDER ADULTS WITH DISABILITIES

**DOI:** 10.1093/geroni/igae098.4127

**Published:** 2024-12-31

**Authors:** Megan Nickrent, Haseeb Rehman, Bethanie Sharp, Lyndsie Koon, Wendy Rogers

**Affiliations:** University of Illinois Urbana-Champaign, Champaign, Illinois, United States; University of Illinois Urbana-Champaign, Champaign, Illinois, United States; University of Kansas, Lawrence, Kansas, United States; University of Kansas, Lawrence, Kansas, United States; University of Illinois Urbana-Champaign, Champaign, Illinois, United States

## Abstract

Volunteering is associated with improved health, decreased symptoms of depression, and increased companionship for older adults. Volunteering can empower people with disabilities, and both the volunteer and the recipient of services experience benefits. Unfortunately, older adults with disabilities often experience barriers to participating in volunteering. Understanding those barriers can highlight areas wherein we can facilitate volunteerism for older adults with disabilities and enable their participation in community activities. We performed an archival analysis of data from Aging Concerns, Challenges, and Everyday Solution Strategies study. Older adults aged 60-79 with vision, hearing, or mobility disabilities were asked about challenges they faced and their responses to those challenges. They provided detailed information in an interview across a range of daily activities, discussing in-depth those that were most difficult. Across the three disability groups, 17 participants discussed difficulty volunteering. Common challenges included organizers of the event not providing accommodations, supervisors not knowing what tasks to assign to persons with disabilities, and other people doubting the abilities of persons with disabilities. Participants expressed frustration with these barriers to volunteering. We explored their responses to these barriers as well. Many participants reportedly used tools or sought assistance from others, but some responded by simply not participating in the activity. Understanding the range of challenges experienced by older adults with disabilities when engaging in the community and exploring their current solutions can help determine ways to increase volunteer engagement for this population. In turn, this can help improve quality of life for older adults with disabilities.

